# Social Network Analysis of COVID-19 Public Discourse on Twitter: Implications for Risk Communication

**DOI:** 10.1017/dmp.2020.347

**Published:** 2020-09-10

**Authors:** Paola Pascual-Ferrá, Neil Alperstein, Daniel J. Barnett

**Affiliations:** Communication Department, Loyola University Maryland; Department of Environmental Health & Engineering, Johns Hopkins Bloomberg School of Public Health

**Keywords:** COVID-19, risk communication, social media, social network analysis, World Health Organization (WHO)

## Abstract

**Objectives:**

The purpose of this study was to demonstrate the use of social network analysis to understand public discourse on Twitter around the novel coronavirus disease 2019 (COVID-19) pandemic. We examined different network properties that might affect the successful dissemination by and adoption of public health messages from public health officials and health agencies.

**Methods:**

We focused on conversations on Twitter during 3 key communication events from late January to early June of 2020. We used Netlytic, a Web-based software that collects publicly available data from social media sites such as Twitter.

**Results:**

We found that the network of conversations around COVID-19 is highly decentralized, fragmented, and loosely connected; these characteristics can hinder the successful dissemination of public health messages in a network. Competing conversations and misinformation can hamper risk communication efforts in a way that imperil public health.

**Conclusions:**

Looking at basic metrics might create a misleading picture of the effectiveness of risk communication efforts on social media if not analyzed within the context of the larger network. Social network analysis of conversations on social media should be an integral part of how public health officials and agencies plan, monitor, and evaluate risk communication efforts.

Most primers on risk communication emphasize the need for public health officials and health agencies to communicate information in a timely, consistent, and clear manner as an effective strategy for disease prevention and preparedness.^1,2^ According to the World Health Organization (WHO), risk communication is essential to help people understand how to protect themselves, stop the spread of disease, and limit the social and economic impact of an outbreak.^3-5^ Risk communication disseminated by public health officials and agencies, however, is not received in a vacuum; rather, it becomes part of a larger ecosystem that includes information from multiple sources, such as friends, family, media, opinion leaders and influencers, among others. Social media, in particular, plays an important role in successfully communicating risk to the larger public. Twitter, in particular, is seen as the leading public communication platform for world leaders^6^ and an essential medium for disseminating public health information during outbreak situations and pandemics.^7-14^ A 2018 Pew Research Center study found that 71% of Twitter users in the United States get their news from Twitter.^15^

Studies looking at how pandemic-related information spreads on Twitter found that while users tend to favor reputable sources of information, many will share information lacking in sound scientific evidence.^11,12^ Conflicting messages from multiple sources of information can lead to increased confusion, higher levels of anxiety and additional negative impacts on mental health, not to mention misguided health behaviors, among the public.^16-21^ They may also increase the likelihood that individuals will act driven by fear rather than by medical guidance from health authorities, and increase skepticism in information sources.^22-24^ Furthermore, mismanagement of risk communication can decrease the credibility of the public health officials and agencies whom people trust with public health and safety.^25^

Monitoring public discourse on social media during a pandemic situation is critical to evaluate the effectiveness of risk communication efforts.^14^ Understanding what topics and messages are driving the conversation and who are mediating conversations is one way of evaluating their impact.^26^ Recent research examining discourse on Twitter around coronavirus disease 2019 (COVID-19) has focused on extracting themes from tweets mentioning “Coronavirus” and “COVID-19,”^27^ analyzing the most-liked tweets made by world leaders^28^ and looking at the spread of information and news frames of top shared sources.^29^ On Twitter, the most visible quantitative metrics to measure the impact of a tweet include the number of replies, retweets, likes, mentions, impressions, reach, and use of a specific #hashtag. Qualitative monitoring includes doing sentiment analysis of tweets, comments, and replies. The latter, however, tend to focus on the actions of individual users and offer a partial view of the impact of communication in a network. A social network approach, on the other hand, can provide a bird’s eye view of public discourse online. Social network analysis (SNA) uses graph theory to represent the structure, makeup, and interaction between members of a network.^30,31^ When applied to conversations on social media, SNA is useful for understanding the impact risk communication can have on a network.

Successful risk communication requires that public health officials and agencies lead the conversation and ensure that the public receives accurate, science-based information when it is most critical. Given the importance of coordinated communications during a pandemic situation,^3-5^ a consistent and unified message that is amplified or reiterated across a network may help increase public awareness about ways to slow the spread and reduce the impact of a virus. The alternative, the fragmentation or atomization of public discourse into clusters of smaller, potentially competing conversations, can dilute the message and reduce the effectiveness of risk communication efforts. Nahon-Serfaty defined this fragmentation of discourses in health communication as “a complex dynamic nourished by competitive and opposite views about diseases’ causes and risk factors, preventive measures, and therapeutic solutions, in the context of globalized media and hyper-information.”^32^ Fragmented discourse, especially when it distracts or misguides the public from what they must do to protect themselves and others, has the potential to worsen the impact of a pandemic.

The purpose of this study was to demonstrate the use of SNA to understand public discourse on Twitter around the novel coronavirus (COVID-19). We focused on key public statements (“communication events”) made by the WHO and its Director-General Dr. Tedros Ghebreyesus during the pandemic. As the leading international organization in the management of pandemics, we predicted that the accounts for the WHO (@WHO) and Dr. Ghebreyesus (@drtedros) would be leading the conversations about the coronavirus on Twitter. We expected the network to be highly centralized, with @WHO and @drtedros at the center of the conversation. Because networks are not static and public discourse on Twitter evolves, we expected the diameter and the modularity measures of the network to increase as more people tweeted about the issue and the discussion became more fragmented. Specifically, we had the following research questions: (1) What is the location of @WHO and @drtedros in the network of conversations around the coronavirus? Specifically, do they appear as central or core opinion leaders?; (2) Who are other opinion leaders on this topic on Twitter?; (3) How has the network changed across time, if at all?; (4) What inferences can we draw from the network properties regarding the successful transmission and adoption of public health messages on Twitter?

## METHODS

For this study, we focused on conversations on Twitter around the novel coronavirus (COVID-19) from late January to early June of 2020. We used Netlytic (https://netlytic.org/), a Web-based software that collects publicly available data from social media sites, such as Twitter, and helps researchers build, visualize, and analyze communication networks using SNA.^33,34^ We focused on 3 key communication events: (1) the WHO’s announcement of the novel coronavirus as a Public Health Emergency of International Concern (PHEIC) on January 30, (2) the WHO’s declaration of COVID-19 as a pandemic on March 11, and (3) the WHO’s updated assessment of the pandemic as a continued global threat on June 8, after surpassing 7 million cases and 400,000 deaths worldwide. For the first event, we used the search term “coronavirus” (the official naming of COVID-19 occurred on February 11). For the second and third events, we used the search terms “coronavirus” and “#COVID19,” a widely used hashtag, to collect tweets containing these terms. We set Netlytic to automatically collect tweets every 15 minutes on the dates specified. Every instance of data collection returned a maximum of 1000 tweets. The data collection generated 3 separate datasets of 100,000 tweets each, which is the maximum amount of records that Netlytic allows per dataset for data visualizations.

We examined the name network to determine the location of @WHO, @drtedros, and other actors in the network. Netlytic defines a name network as “a communication network built from mining personal names in the messages.”^35^ Netlytic will extract each @name mentioned in a tweet and plot them in the network graph as separate nodes with edges or ties connecting the message author @name to each of the @names (s)he/they mentioned in the tweet. Twitter is a directed network, which means network visualizations of Twitter data will show a tie or an edge when a user mentions or replies to another user, not if they are following each other. That is different from Facebook, which is an undirected network and would show connections between “friends” as ties or edges.

For the social network visualizations, we used the Distributive Recursive Layout (DrL), which is “a force-directed graph layout, effective for visualizing large networks.”^35^ In this layout, long edges are hidden to highlight clusters or communities of conversation. Clusters are groups of nodes that share a particular characteristic (eg, geographic location, sub-topic, or theme). These communities appear on the graph as round or oval shapes. The DrL layout is well-suited for visualizing all the different conversations happening over time and identifying who is at the center of each conversation. In some cases, the conversations are unrelated, with the only commonality being the search term or hashtag used. We examined diameter, density, reciprocity, centralization, and modularity to understand the topology of the network. We inspected who was mentioned the most, who posted the most, and who were retweeted the most to assess influence.

Finally, we also wanted to examine different network properties that might affect the successful dissemination and adoption of public health messages. Network properties include measures such as diameter, density, reciprocity, centralization, and modularity.^35^ The diameter measures the longest distance between 2 users in the network, counted in the number of nodes or unique Twitter user accounts (@name), that it takes to get from 1 participant to the other. Density measures how close nodes are in a network, while reciprocity measures 2-way communication or how much nodes are talking to each other. Centralization measures the extent to which a few nodes dominate the conversation. Each node has a centrality measure: indegree (based on times it has been mentioned or replied to), outdegree (based on times it has mentioned or replied to others), and total degree (the sum of both). Finally, modularity measures the fragmentation of a network into distinct communities. For all of these measures, values range from 0 (lowest) to 1 (highest). For example, modularity values closer to 1 “indicate clear divisions between communities,” whereas values less than 0.5 suggest that the communities “overlap more; the network is more likely to consist of a core group of nodes.”^35^ A high modularity value reflects greater separation between communities of conversation. A network that is low in centrality means that there are many moderators and opinion leadership is less centralized, making it harder for public health officials and agencies to lead the conversation or have control of the message. Networks that are close-knit and homophilous, where people are similar to each other, tend to facilitate public health information dissemination and adoption of health behaviors.^36,37^ On the other hand, heterogeneous networks that are more spread out (larger in diameter), loosely connected (low in density and reciprocity), and fragmented (high in modularity) could lead to slower diffusion of information and threaten widespread adoption of scientifically sound health recommendations.

## RESULTS

### Communication Event 1: WHO Declares the Novel Coronavirus a Public Health Emergency of International Concern

**FIGURE 1:**
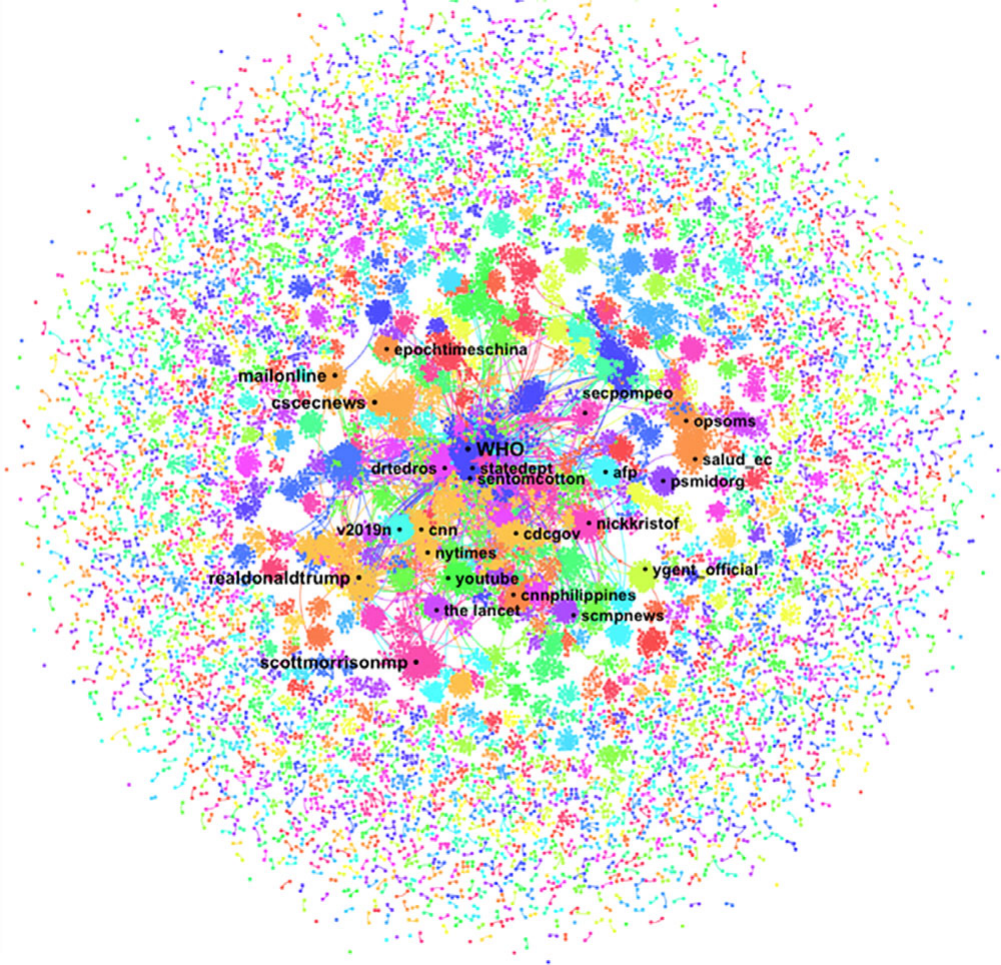
Name Network (DrL) for the Term “Coronavirus” With the Top Names Mentioned in the Description Field of Tweets Collected from January 30, 2020, at 7:53 am (AST) to January 31, 2020, at 8:54 am (AST). We used Netlytic to generate this network visualization.

One-hundred thousand tweets including “coronavirus” by 89,690 unique posters were collected from January 30, 2020, at 7:53 am (AST) to January 31, 2020, at 8:54 am (AST). The DrL visualization (Figure 1) shows the nodes with the highest total degree centrality in a network of 13,872 posters with 24,113 ties (including self-loops). A total of 97,920 unique @names were mentioned or replied to within the tweets. The top 5 nodes mentioned were @WHO (*N* = 1985), @drtedros (*N* = 348), @youtube (*N* = 268), @realdonaldtrump (*N* = 217), @cdcgov (*N* = 119), and @cnn (*N* = 100). The network diameter is 7 nodes. The density measure (*N* = 0.00), the reciprocity measure (*N* = 0.00), and the centralization measure (*N* = 0.04) are all low. The modularity measure (*N* = 0.94) is high. Six of the top posters, defined as those users who generated the greatest number of tweets with the term “coronavirus” are accounts that self-identify as a bot or as automated in their profile bio. Approximately 81% of messages in the network were direct retweets (RTs). The users most retweeted were @helloalegria (*N* = 2023), @spectatorindex (*N* = 1093), @solineura (*N* = 845), @zornitsaxx (*N* = 837), and @realdonaldtrump (*n* = 787).

### Communication Event 2: WHO Declares COVID-19 a Pandemic

**FIGURE 2:**
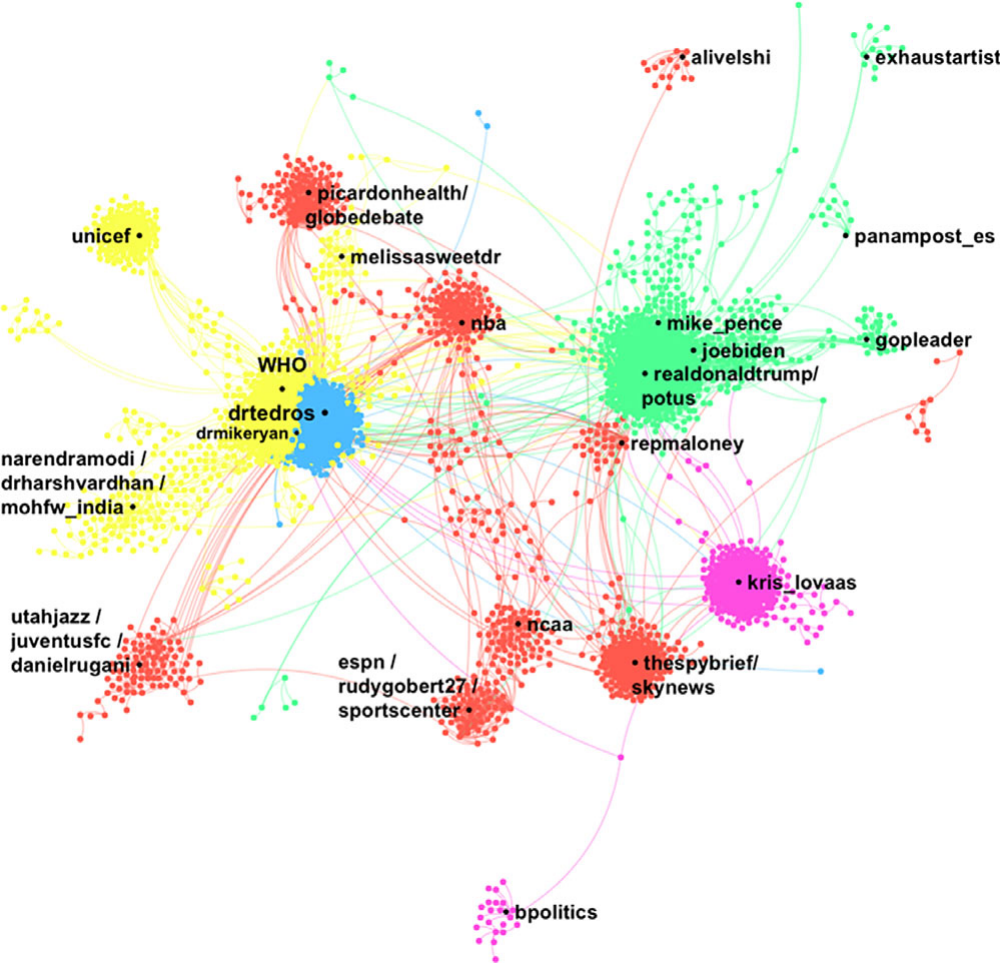
Name Network (DrL) for the Term “Coronavirus” and “#COVID19,” Showing Only the 5 Main Clusters With the Top Names Mentioned in the Description Field of Tweets Collected From March 11, 2020, at 13:28 pm (AST) to March 12, 2020, at 1:54 am (AST). We used Netlytic to generate this network visualization.

One-hundred thousand tweets including “coronavirus” and “#COVID-19” by 89,176 unique posters were collected from March 11, 2020, at 1:28 pm (AST) to March 12, 2020, at 1:54 am (AST). The DrL visualization (Figure 2) shows the nodes with the highest total degree centrality in a network of 22,306 posters with 36,509 ties (including self-loops). A total of 97,209 unique @names were mentioned or replied to within the tweets. The top 5 nodes mentioned were @drtedros (*N* = 7574), @realdonaldtrump (*N* = 1845), @WHO (*N* = 1652), @kris_lovaas (*N* = 693), @potus (*N* = 508), and @thespinofftv (*N* = 419). The network diameter is 24 nodes. The density measure (*N* = 0.00), the reciprocity measure (*N* = 0.00), and the centralization measure (*N* = 0.09) are all low. The modularity measure (*N* = 0.90) is high. Four of the top posters are accounts that self-identify as news aggregators in their profile bio, 2 self-identify as bots, and 4 seem to be individual users who have a high retweet ratio or post very frequently. Approximately 86% of the records in the dataset represented RTs. The users most retweeted were @WHO (*N* = 8988), @conflits_fr (*N* = 2782), @drdenagrayson (*N* = 2,165), @noticiasonu (*N* = 1,629), and @feelthepress (*N* = 795).

### Communication Event 3: WHO Warns Countries That COVID-19 Continues to Be a Global Threat, Urges Them to Continue to Fight It Actively and not to Let Their Guard Down

**FIGURE 3:**
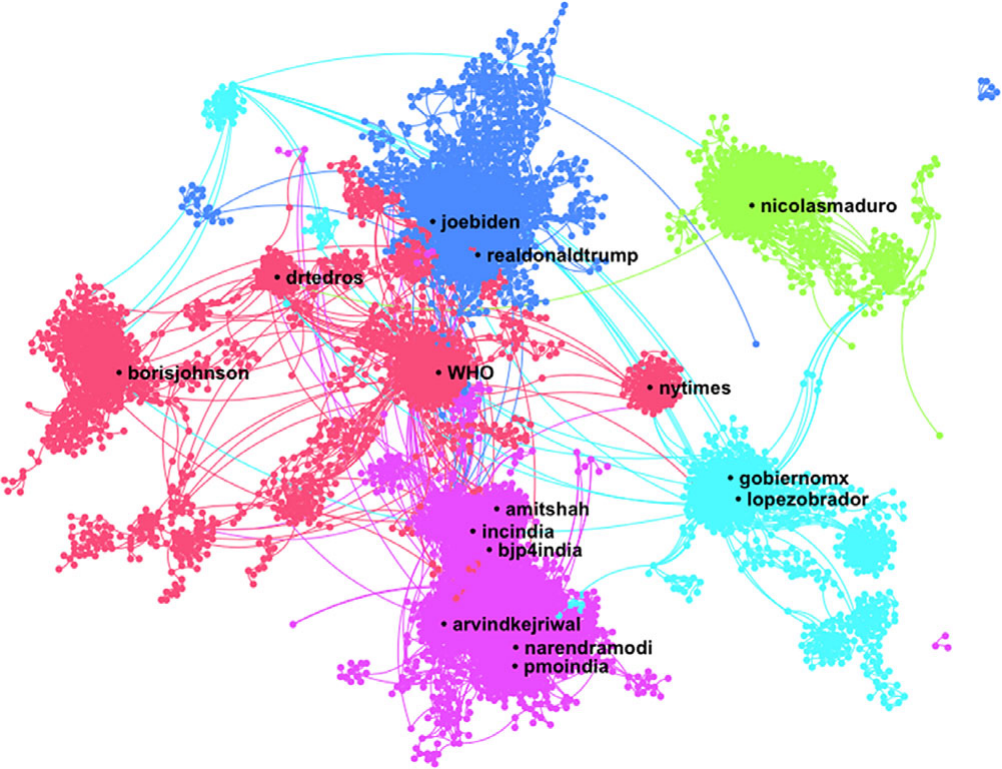
Name Network (DrL) for the Term “Coronavirus” and “#COVID19,” Showing Only the 5 Main Clusters With the Top Names Mentioned in the Description Field of Tweets Collected From June 8, 2020, at 19:58 pm (AST) to June 9, 2020, at 20:24 pm (AST). We used Netlytic to generate this network visualization.

One-hundred thousand tweets including “coronavirus” and “#COVID19” by 90,014 unique posters were collected from June 8, 2020, at 7:58 pm (AST) to June 9, 2020, at 8:24 pm (AST). The DrL visualization (Figure 3) shows the nodes with the highest total degree centrality in a network of 25,295 posters with 52,031 ties (including self-loops). A total of 111,069 unique @names were mentioned or replied to within the tweets. The top 5 account names mentioned were @realdonaldtrump (*N* = 914), @secretarycarson (*N* = 636), @nicolasmaduro (*N* = 346), @lopezobrador (*N* = 342), and @WHO (*N* = 340). Tweets mentioning @secretarycarson are all retweets of @realdonaldtrump thanking him.^38^ The network diameter is 25 nodes. The density measure (*N* = 0.00), the reciprocity measure (*N* = 0.00), and the centralization measure (*N* = 0.01) are all low. The modularity measure (*N* = 0.96) is high. The top posters included a merchandise store, 3 news organizations or news aggregators, a journalist, a freelance writer, an entertainment podcast, and an individual account with a high retweet ratio. Approximately 78% of the records in the dataset represented RTs. The users most retweeted were @kyn_joy (*N* = 1003), @youranoncentral (*N* = 761), @drjcofthedc (*N* = 716), @realdonaldtrump (*N* = 635), and @reaganschmagan (*N* = 438).

## DISCUSSION

While @WHO and @drtedros had the highest total degree centrality in the first 2 events, the networks themselves were highly decentralized. For the first event, @WHO was the most mentioned name in the network within the description field of tweets, followed by @drtedros. For the second event, @drtedros was the most mentioned name, followed by @realdonaldtrump and @WHO. This is not surprising given the importance of the WHO declaring the novel coronavirus a PHEIC on January 30 and a pandemic on March 11. The press conferences held by Dr. Ghebreyesus and the WHO were successful in attracting the attention of Twitter users following the coronavirus. What is interesting is the increasing attention earned by government figures as the pandemic continued to evolve. For the third event, @realdonaldtrump was the most mentioned name in the network, followed by other government figures from the United States, Venezuela, and Mexico. @WHO appears as the fifth most mentioned name in the network, and @drtedros was absent from the list of the top 30 mentioned names altogether. By early June, conversations around COVID-19 had become much more political than public health-focused, almost leaving @WHO and @drtedros outside of the conversation.

At the same time, the number of mentions must be interpreted in the context of the network. For example, the number of mentions of @WHO represents less than 2% of all messages in the datasets for the first 2 events, and less than 0.5% of all messages for the third event. Mentions of @drtedros during the second event accounted for 7.5% of all messages in the network. The centralization values for all 3 network visualizations were very low (*N* ≤ 0.09), which means the networks were highly decentralized. At the same time, the number of messages mentioning the top @names in each network visualization does not account for a significant portion of the dataset; therefore, it is difficult to label any of the actors as core opinion leaders. Additionally, @names mentioned reflect who people are talking about the most, not who is doing most of the talking. Many of the mentions are negative in sentiment; they appeared to be more critical than supportive of actors’ messages. Because of this, while @WHO and @drtedros received the most attention in the first 2 events, we conclude that the network of conversations about the coronavirus on Twitter is highly fractured and without clear leadership.

Applying the term conversation is also problematic since we found that 78 to 86% of tweets posted during each time frame were direct retweets of messages posted by others. This is consistent with the low reciprocity measures that we got; most people were not having conversations but rather broadcasting information and amplifying others’ messages. Retweets are considered a form of 1-way communication and do not usually lead to conversations. At the same time, retweets allow us to identify whose messages are being amplified the most and can serve as a measure of indirect influence and opinion leadership. When we looked at retweets, some of the actors changed but their level of influence was still small compared with the size of the network. For example, while the most talked-about @names for the first event were @WHO and @drtedros, the most retweeted user was @helloalegria, whose tweet calling out racist behavior toward Chinese people in the context of the coronavirus has been replied to 925 times, retweeted over 82,500 times and liked over 257,400 times.^39^ The tweet was in response to a video post from another user with the caption, “This is RACIST.”^40^ That tweet has been replied to over 2500 times, retweeted over 24,100 times, and liked over 89,100 times. The video itself has more than 5.1 million views. In comparison, while being the top name mentioned in the network for the first event, the most retweeted post from @WHO has been replied to 627 times, retweeted 894 times, and liked over 1300 times since January 30.^41^

The most retweeted post by @drtedros for the second event has been replied to 605 times, retweeted over 4200 times, and liked over 3800 times.^42^ The video included in the post, however, has over 2 million views. In summary, the individual’s tweet commenting on the racialization of the virus got more traction than the official announcement made by Dr. Ghebreyesus and the WHO declaring COVID-19 a pandemic. Our analysis showed that in 2 of the 3 events, public health agencies, which should be leading the risk communication charge, were being second to individual social media influencers. This is concerning given the accompanying misinformation epidemic that has surrounded COVID-19 as evident by recent infodemiological studies in this area.^29,43-47^ The issue of misinformation is one that is worthy of its own study. The WHO defines an infodemic as “an overabundance of information—some accurate and some not—rendering it difficult to find trustworthy sources of information and reliable guidance,” which in turn could have detrimental effects on the population and public health efforts to combat the virus itself.^48^ If public health agencies are to successfully combat the infodemic surrounding COVID-19, then it is critical that they lead the conversation.

We also observed changes in the network structure as the pandemic evolved, although not on all measures. The most significant change observed was in the network diameter, which went from 7 to 25 nodes from the first to the third events. This is not surprising with more people talking about COVID-19. More topics lead to fracturing into clusters or niches within the broader social network that forms around those topics. This, in turn, increases the diameter of the network, particularly when nodes are loosely connected, and people are not necessarily speaking to each other. Centralization was slightly higher for the second event, when WHO declared COVID-19 a pandemic, although the value was still very low. Modularity always remained high, increasing from 0.94 for the first event to 0.99 for the third event, representing increased fragmentation of the network. Density and reciprocity remained at 0.00 throughout all 3 events. There could be several explanations for this. First, the size of the datasets might be too large to get high values of density and reciprocity for the network as a whole, even though there might be individual clusters in the network that are higher in density and reciprocity. Another consideration is whether the topic lends itself to a conversation or is a news item that users are more likely to share with their followers, with commentary or as a direct retweet. Another possible explanation is that many more people may be using Twitter for broadcasting purposes rather than for being social and talking to others.

In addition to @WHO and @drtedros, we found that @realdonaldtrump, the verified personal account for US President Donald Trump, was the other node consistently and prominently mentioned throughout the pandemic, moving from the fourth most mentioned in the first event to the most mentioned in the third event. His tweets concerning the coronavirus were among the most retweeted in the first and third events. However, as discussed above, not all mentions were positive; many of the messages mentioning @realdonaldtrump were negative in sentiment. Of interest, @cdcgov, the verified account of the Centers for Disease Control in the United States, appeared among the top 5 mentioned names on January 30, but not for the other 2 events. Beyond the most mentioned names, some themes emerged in the network visualizations. For the second event, localized conversations included a cluster focusing on US politics, another focused on the impact of COVID-19 on sports, specifically basketball, a media-moderated debate in Canada, and one focused on COVID-19 news in India. For the third event, there are conversations clustered by geographic location in India, Mexico, the United Kingdom, and Venezuela, most of them focusing on government actors. Other than its impact on the world of sports, the conversations around COVID-19 turned political in several countries, not just the United States. This is concerning given that the politicization of a virus threatens the credibility of scientifically based public health information and the effectiveness of public health officials and health agencies worldwide charged with doing risk communication.

Based on previous research studies looking at network topologies, the structure of these networks (highly decentralized, fragmented, and loosely connected) will hinder the successful dissemination of risk communication by public health officials and health agencies across the network. The high measure of modularity shows how fragmented the public’s attention is on this topic. The growing diameter size, paired with low density, low reciprocity and high modularity, make it difficult for a topic to grab everyone’s attention, either because participants do not care, and if they do, they might not feel motivated to comment on or share that content. What seems important from a risk communication standpoint is that looking at basic social media metrics might create a misleading picture of the effectiveness of risk communication efforts on social media if not analyzed within the context of the larger network. Basic social media metrics do not provide that information. SNA of conversations on social media should be an integral part of how public health officials and agencies plan, monitor, and evaluate risk communication efforts.

Our study has some limitations. First, due to Twitter’s application programming interface restrictions, Netlytic limits data collection to 1000 tweets every 15 minutes. In other words, the tweets analyzed do not represent all of the tweets that were posted during the 3 communication events. Second, the low density and reciprocity measures that we saw across all communication events may not be unique to these networks; rather it could be representative of how people view and use Twitter, for news gathering and broadcasting, as opposed to other platforms like Facebook, which lend themselves more to conversations with others. Third, just because users are not engaging with content on Twitter does not mean that they are not commenting, sharing and/or adopting recommendations from public health officials and agencies through other platforms or offline.

## CONCLUSIONS

In this study, we used SNA to examine and understand public discourse on Twitter around 3 key announcements made by the WHO during the COVID-19 pandemic. Specifically, we were interested in demonstrating the use of SNA to understand the network of conversations and actors regarding the novel coronavirus, identify potential roadblocks in the successful dissemination and adoption of health information, and realign risk communication messaging accordingly. We found that the network of conversations around COVID-19 is highly decentralized, fragmented, and loosely connected; these properties can hinder the successful dissemination of public health information. Also, competing conversations, misinformation, and other distractions by politically motivated actors can hamper risk communication efforts by public health officials and health agencies in a way that imperils public health. It is important, then, that public health agencies monitor communication on social media beyond basic quantitative social media metrics and text or content analyses. We recommend the integration of SNA as a best practice in risk communication on social media. This extended view recognizes that the space in which the discussion over COVID-19 takes place is one that is quite varied and includes diffuse users who are not only geographically diverse but also who represent divergent points of view and interest in this subject, ranging from scientific and public health experts to ordinary citizens who are seeking and sharing information to users who intentionally spread disinformation. Doing so will help public health agencies understand who is mediating the discussion regarding COVID-19 and what they can do to fight the accompanying infodemic.
